# Antiinflammatory evaluation of leaves of *Plumeria acuminata*

**DOI:** 10.1186/1472-6882-6-36

**Published:** 2006-11-02

**Authors:** M Gupta, UK Mazumder, P Gomathi, V Thamil Selvan

**Affiliations:** 1 Department of Pharmaceutical Technology, Jadavpur University, Kolkata, West Bengal, India; 2 Department of Pharmaceutical Chemistry, ASN Pharmacy college, Burripalem road, Nelapadu, Tenali, Andhra Pradesh, India

## Abstract

**Backround:**

*Plumeria acuminata *belonging to the family Apocynaceae is commonly known as '*perungalli*' in Tamil and is widely distributed throughout the Southern parts of India. In traditional medicinal system different parts of the plant have been mentioned to be useful in a variety of diseases. The plant material is widely used as a purgative, remedy for diarrhoea and cure for itch. The milky juice is employed for the treatment of inflammation and rheumatism. The bark has been reported to be useful in hard tumors, diarrhoea and gonorrhoea. The objective of the present study was to evaluate the antiinflammatory activity of methanol extract of leaves of *Plumeria acuminata *on carrageenan, dextran, histamine and serotonin-induced inflammation in rat hind paw oedema models.

**Methods:**

Acute and chronic inflammation models were used to evaluate the anti-inflammatory activity of the extract. Wistar albino rats of either sex weighing 180–200 g were used. In acute model carrageenan, dextran, histamine and serotonin models were used to induce inflammation in rat hind paw and cotton pellet-induced granuloma method was used for chronic inflammation model. In each model four groups of six animals were used. In all the models Group I served as control (0.9% normal saline, 5 mlkg^-1 ^b.w) and group IV as standard (Indomethacin 10 mgkg^-1 ^b.w). Group II and III received extract at the doses of 250 and 500 mgkg^-1 ^b.w respectively.

**Results:**

The methanol extract of *Plumeria acuminata *exhibited significant anti-inflammatory activity on the tested experimental animal models. The extract (500 mgkg^-1 ^b.w) exhibited maximum antiinflammatory effect i.e., 30.51, 47.06, 34.48 and 32.50% (P < 0.001) at the end of 3 h with carrageenan, dextran, histamine and serotonin respectively. Administration of MEPA (500 mgkg^-1 ^b.w) and indomethacin (10 mgkg^-1 ^b.w) significantly reduced the formation of granuloma tissue induced by cotton pellet method at a rate of 45.06 and 51.57% respectively. The effect produced by the extract was comparable to that of indomethacin a prototype of a nonsteroidal antiinflammatory agent.

**Conclusion:**

The results obtained in this study indicated that the methanol extract of *Plumeria acuminata *possess potent antiinflammatory activity in both acute and chronic models.

## Background

In Indian system of medicine, a large number of drugs of either herbal or mineral origin have been advocated for various types of diseases and other different unwanted conditions in humans [1]. Ayurveda is one of the traditional systems of medicine practiced in India and Sri Lanka and can be traced back to 6000 BC [2]. Ayurvedic medicines are largely based upon herbal and herbomineral preparations and have specific diagnostic and therapeutic principles [3].

Inflammation is a disorder involving localized increases in the number of leukocytes and a variety of complex mediator molecules [4]. Prostaglandins are ubiquitous substances that indicate and modulate cell and tissue responses involved in inflammation. Their biosynthesis has also been implicated in the pathophysiology of cardiovascular diseases, cancer, colonic adenomas and Alzheimer's disease [5,6].

Medicinal plants are believed to be an important source of new chemical substances with potential therapeutic effects [7,8]. The research into plants with alleged folkloric use as pain relievers, antiinflammatory agents, should therefore be viewed as a fruitful and logical research strategy in the search for new analgesic and antiinflammatory drugs [9].

*Plumeria acuminata *belonging to the family Apocynaceae is commonly known as '*perungalli*' in Tamil and widely distributed throughout the Southern parts of India. In traditional medicinal system different parts of the plant have been mentioned to be useful in a variety of diseases. The plant material is widely used as a purgative, remedy for diarrhoea and cure for itch. The milky juice is employed for the treatment of inflammation and rheumatism. The bark has been applied as a plaster over inflammation and hard tumors. The leaves are reported to have antiinflammatory, rubefacient in rheumatism and have strong purgative effect. Its branches are used like those of '*chitraka' *to produce abortion [10]. However there is no scientific report or verification of the use of this plant in the treatment of these conditions. Accordingly a pharmacological investigation on the methanol extract of leaves of *Plumeria acuminata *(MEPA) has been initiated in our laboratory and here we report the preliminary result of studies on acute toxicity and antiinflammatory effects on experimental models.

## Methods

### Plant material

The leaves of the plant *Plumeria acuminata *(Family: Apocynaceae) was collected from Erode district of Tamilnadu, India. The plant material was taxonomically identified by Botanical Survey of India, Kolkata. A voucher specimen (No. GMG 02/05) has been preserved in our laboratory for future reference. The leaves were dried under shade and then powdered with a mechanical grinder and stored in airtight container. The dried powder material of the leaves was defatted with petroleum ether and the marc thus obtained was then extracted with methanol in a soxhlet apparatus. The solvent was completely removed under reduced pressure and a semisolid mass was obtained (MEPA, yield 12.4%). The dried MEPA was suspended in normal saline and used for the present study.

### Drugs

Carrageenan (S.D. Fine Chemicals Limited, Bombay), 5-hydroxytryptamine hydrochloride (Serotonin), histamine and dextran (Sigma, USA) were used in the study and indomethacin (Recon, Bangalore) was used as the standard drug.

### Animals

Studies were carried out using Wistar albino rats of either sex weighing 180–200 g and Swiss albino mice of either sex weighing 18–22 g. They were obtained from the animal house, Indian Institute of Chemical Biology (IICB), Kolkata, India. The animals were grouped in polyacrylic cages (38 cm × 23 cm × 10 cm) with not more than six animals per cage and maintained under standard laboratory conditions (temperature 25 ± 2°C) with dark and light circle (14/10 h). They were allowed free access to standard dry pellet diet (Hindustan Lever, Kolkata, India) and water *ad libitum*. The rats were acclimatized to laboratory condition for 10 days before commencement of experiment. All procedures described were reviewed and approved by the university animal ethical committee.

### Phytochemical screening

The extract was screened for the presence of various constituents employing standard screening test [11]. Conventional protocol for detecting the presence of steroids, alkaloids, tannins, flavonoids, glycosides, etc., was used.

### Acute toxicity test

The animals were divided into six groups containing eight animals in each group. MEPA was suspended in normal saline and administered orally as a single dose to groups of mice at different concentrations (500, 750, 1000, 1250, 1500 and 2000 mgkg^-1 ^b.w). These animals were observed for a 72 h period. The number of deaths was expressed as a percentile and the LD_50 _was determined by probit a test using the death percentage versus the log dose [12].

### Antiinflammatory activity

#### Carrageenan-induced rat paw oedema

The rats were divided into four groups (n = 6). The different groups were treated orally with MEPA (250 and 500 mgkg^-1 ^b.w), indomethacin (10 mgkg^-1 ^b.w), and vehicle control (0.9% NaCl, 5 mlkg^-1 ^b.w). The administration of extract and drugs was 30 min prior to injection of 0.1 ml of 1% freshly prepared suspension of carrageenan in normal saline in the right hind paw subplantar of each rat. The paw volume was measured initially and then at 1, 2, 3 and 4 h after the carrageenan injection by using plethysmometer [13]. The antiinflammatory effect of MEPA was calculated by the following equation:- Antiinflammatory activity (%) = (1-D/C) × 100, where D represents the percentage difference in paw volume after the administration of drugs to the rats and C represents the percentage difference of volume in the control groups [14].

#### Dextran-induced rat paw oedema

The animals were treated in a manner similar to that of carrageenan-induced paw oedema models; dextran (0.1 ml, 1% w/v in normal saline) was used in the place of carrageenan [13].

#### Histamine-induced rat paw oedema

In this model hind paw oedema in the right foot of a rat was induced by subplantar injection of 0.1 ml of 1% freshly prepared histamine in normal saline and the paw oedema was measured as mentioned earlier [14].

#### Serotonin-induced rat paw oedema

In another model oedema of the right hind paws of the rat was induced by subplantar injection of 0.1 ml of 1% freshly prepared serotonin in normal saline. Group division and treatment of the animals were the same as the Carrageenan-induced rat paw oedema model and the paw volume was measured as mentioned in Winter et al., 1962 [15].

#### Cotton pellet-induced granuloma

The cotton pellets-induced granuloma in rats was studied according to the method D'Arcy et al., 1960 [16]. The animals were divided into four groups of six animals in each group. The rats were anaesthetized and sterile cotton pellets weighing 10 ± 1 mg were implanted subcutaneously into both sides of the groin region of each rat. Group I served as control and received the vehicle (0.9% NaCl, 5 mlkg^-1 ^b.w. The extract MEPA at the concentration of 250 and 500 mgkg^-1 ^b.w was administered orally to groups II and III animals for seven consecutive days from the day of cotton pellet implantation. Group IV animals received indomethacin at a dose of 10 mgkg^-1 ^b.w for the same period. On 8^th ^day the animals were anaesthetized and the pellets together with the granuloma tissues were carefully removed and made free from extraneous tissues. The wet pellets were weighed and then dried in an oven at 60°C for 24 h to constant weight, after that the dried pellets were weighed again. Increment in the dry weight of the pellets was taken as a measure of granuloma formation. The antiproliferative effect of MEPA was compared with control.

### Statistical analysis

The values were expressed as mean ± S.E.M. The statistical significance was determined by using the student *t-*test [17]. Values of P < 0.001 were considered as statistically significant.

## Results

### Phytochemical screening

Preliminary phytochemical screening of the methanol extract revealed the presence of steroids, flavonoids, tannins, alkaloids and glycosides. Further separation of the specific phytochemical is in progress.

### Acute toxicity test

In the acute toxicity assay no deaths were observed during the 72 h period at the doses tested. At these doses, the animals showed no stereotypical symptoms associated with toxicity, such as convulsion, ataxy, diarrhoea or increased diuresis. The median lethal dose (LD_50_) was determined to be higher than highest dose tested i.e., 2.0 gkg^-1 ^b.w.

### Carrageenan-induced rat paw oedema

**Figure 1:**
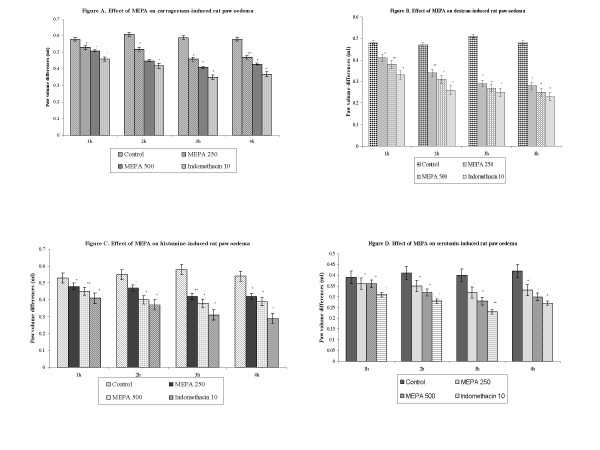
**(A)** Effect of MEPA on carrageenan-induced rat paw oedema.** (B)** Effect of MEPA on dextran-induced rat paw oedema.** (C)** Effect of MEPA on histamine-induced rat paw oedema.** (D)** Effect of MEPA on serotonin-induced rat paw oedema. In all the graphs. Values are mean ± S.E.M. (n = 6). Variation compared to the control animals. * (P < 0.001) ** (P < 0.01).

The antiinflammatory activity of MEPA was measured at the dose of 250 and 500 mgkg^-1 ^b.w against acute paw oedema induced by carrageenan is summarized in Figure 1. The MEPA produced significant (P < 0.001) antiinflammatory activity and the results were comparable to that of indomethacin as a standard antiinflammatory drug. MEPA at the doses 250 and 500 mg/kg moderately inhibited (22.03 and 30.51%) the carrageenan-induced rat paw oedema and it has less activity compared to other models.

### Dextran-induced paw oedema

The differences in the paw volume after the administration of MEPA and standard drug indomethacin were presented in Figure 1b. The extract produced significant antiinflammatory activity and the results were comparable to that of the standard drug indomethacin. MEPA exhibited 43.14 and 47.06% of inhibition at the dose of 250 and 500 mgkg^-1 ^b.w respectively in dextran-induced paw oedema in rats.

### Histamine and serotonin-induced paw oedema

The antiinflammatory effect of MEPA against acute pedal oedema induced by phlogistic agents histamine and serotonin has been shown in Figures 1c and 1d. MEPA showed significant antiinflammatory activity and the results were comparable to that of indomethacin a prototype of a non-steroidal antiinflammatory drug. The extract (500 mgkg^-1^) showed a maximum 34.48% inhibition in histamine and 32.50% inhibition in serotonin-induced rat paw oedema.

### Cotton pellets-induced granuloma

The effects of MEPA and indomethacin on the proliferative phase of inflammation are shown in table 1. A significant reduction in the weight of cotton pellets was observed with MEPA (250 and 500 mgkg^-1 ^b.w) compared to the vehicle treated rats. However the degree of reduction was less than the effect caused by indomethacin.

**Table 1:** Effect of MEPA on cotton pellets-induced granuloma in rats

Treatment	Dose (mg/kg)	Weight of cotton pellets (mg) (wet)	Percentage inhibition	Weight of cotton pellets (mg) (dry)	Percentage inhibition
Control (0.9% NaCl)	5 ml	183.17 ± 14.3	-	48.62 ± 3.6	-
MEPA	250	101.64 ± 9.6*	44.51	32.36 ± 2.3	33.43
MEPA	500	87.47 ± 7.4*	52.25	26.71 ± 2.1*	45.06
Indomethacin	10	78.25 ± 6.3*	57.28	23.54 ± 2.4*	51.57

Values are mean ± S.E.M. (n = 6).

* Experimental groups were compared with control (P < 0.01).

## Discussion and conclusion

The present study establishes the antiinflammatory activity of the methanol extract of the leaves of *Plumeria acuminata *in the models used. Using acute toxicity assay, the median lethal dose, LD_50 _was determined to be higher than 2.0 gkg^-1 ^b.w. In this assay, neither deaths nor symptoms associated with toxicity such as convulsion, ataxy, diarrhoea or increased diuresis occurred during the 72 h observation period. These results indicate the effectiveness and relative safety of MEPA for the treatment of conditions associated with inflammation.

Carrageenan-induced oedema has been commonly used as an experimental animal model for acute inflammation and is believed to be biphasic. The early phase (1 – 2 h) of the carrageenan model is mainly mediated by histamine, serotonin and increased synthesis of prostaglandins in the damaged tissue surroundings. The late phase is sustained by prostaglandin release and mediated by bradykinin, leukotrienes, polymorphonuclear cells and prostaglandins produced by tissue macrophages [18]. The inhibitory activity shown by the extract of *Plumeria acuminata *leaves (250 and 500 mgkg^-1 ^b.w) over a period of 4 h in carrageenan, dextran. Serotonin and histamine-induced paw inflammation was quite similar to that exhibited by the group treated with indomethacin. These results indicate that the extract acts in later phases probably involving arachidonic acid metabolites, which produce an oedema dependent on neutrophils mobilization [19].

The cotton pellet method is widely used to evaluate the transudative and proliferative components of the chronic inflammation. The wet weight of the cotton pellets correlates with the transuda; the dry weight of the pellets correlates with the amount of the granulomatous tissue [20,21]. Administration of MEPA (250 and 500 mgkg^-1 ^b.w) and indomethacin (10 mgkg^-1 ^b.w) appear to be effective in inhibiting the wet weight of cotton pellet. On the other hand, the MEPA effect on dry weight of the cotton pellet was almost near to that of indomethacin. These data support the hypothesis of the greater effect of the MEPA on the inflammation in rats. This effect may be due to the cellular migration to injured sites and accumulation of collagen an mucopolysaccharides.

Based on the results it can be concluded that the methanolic extract of leaves of *Plumeria acuminata *possess an antiinflammatory property in both acute and chronic phases of inflammation.

## Competing interests

The author(s) declare that they have no competing interests.

## Authors' contributions

MG participated in the animal experiment and drafted the manuscript. UKM performed the statistical analysis. PG carried out the experimental work. VT participated in this experimental design and coordination.

## Pre-publication history

The pre-publication history for this paper can be accessed here:


